# Modernising epidemic science: enabling patient-centred research during epidemics

**DOI:** 10.1186/s12916-016-0760-x

**Published:** 2016-12-19

**Authors:** Amanda M. Rojek, Peter W. Horby

**Affiliations:** 1 Centre for Tropical Medicine and Global Health, Nuffield Department of Medicine, University of Oxford, Old Road Campus, Roosevelt Drive, Oxford, OX3 7FZ UK; 2 International Severe Acute Respiratory and emerging Infections Consortium, University of Oxford, Oxford, UK

**Keywords:** Epidemic, Pandemic, Outbreak, Clinical research, Clinical trial, Ebola, Zika

## Abstract

**Background:**

Emerging and epidemic infectious disease outbreaks are a significant public health problem and global health security threat. As an outbreak begins, epidemiological investigations and traditional public health responses are generally mounted very quickly. However, patient-centred research is usually not prioritised when planning and enacting the response. Instead, the clinical research response occurs subsequent to and separate from the public health response, and is inadequate for evidence-based decision-making at the bedside or in the offices of public health policymakers.

**Discussion:**

The deficiencies of the clinical research response to severe acute respiratory syndrome, pandemic influenza, Middle East respiratory syndrome coronavirus and Ebola virus demonstrate that current research models do not adequately inform and improve the quality of clinical care or public health response. Three suggestions for improvements are made. First, integrate the data and sample collection needs for clinical and public health decision-making within a unified framework, combined with a risk-based, rather than a discipline-based, approach to ethical review and consent. Second, develop clinical study methods and tools that are specifically designed to meet the epidemiological and contextual challenges of emerging and epidemic infectious diseases. Third, invest in investigator-led clinical research networks that are primed and incentivised to respond to outbreak infections, and which can call on the support and resources of a central centre of excellence.

**Conclusions:**

It is crucial that the field of epidemic science matures to place patients at the heart of the response. This can only be achieved when patient-centred research is integrated in the outbreak response from day one and practical steps are taken to reduce the barriers to the generation of reliable and useful evidence.

## Background

Emerging and epidemic infectious diseases (EEIDs) have shaped society, and recent events affirm that they will continue to do so. In less than two years, Ebola virus disease (EVD) and the Zika virus prompted the World Health Organization (WHO) to declare Public Health Emergencies of International Concern. Meanwhile, Middle East respiratory syndrome coronavirus (MERS-CoV) continues to cause sporadic cases and nosocomial outbreaks, and an increasing diversity of avian influenza viruses are infecting people across numerous continents [1, 2]. With the Commission on Creating a Global Health Risk Framework for the Future estimating that the annual cost of a potential pandemic is around US $60 billion [3], epidemic infectious diseases remain a force to be reckoned with [4].

Preparing ourselves adequately for these threats demands action on many fronts, including the strengthening of health systems, improved surveillance and response capabilities, and better pipelines for developing diagnostics, therapeutics and vaccines. ‘Patient-centred’ research needs to be included as one key pillar of an enhanced outbreak investigation, response and control system. Patients are the primary source of much of the information (e.g. clinical presentation and outcomes) and materials (e.g. pathogens and antibodies) that is vital for both clinical and public health decision-making; for advancing basic scientific understanding; and for evaluating the products of enhanced diagnostic, drug and vaccine development pipelines. We argue that our thinking should therefore converge on the patient and we should address the needs of all disciplines within a strengthened and unified framework.

### The importance of patient-centred research during epidemics

#### Improving patient outcomes

In the turmoil of epidemics and the pressure to protect public health and economic interests, it is sometimes forgotten that patients lie at the heart of every outbreak. These patients, their families and the clinical teams caring for them are often struggling with frightening uncertainty and inadequate support and resources. However, during epidemics, decisions such as which drugs, fluids or supportive care strategies to offer patients are usually made on an ad hoc basis by the treating clinician, or from guidelines that approximate from other diseases and experiences [5, 6]. The African trial of Fluid Expansion As Supportive Therapy (FEAST) for critically ill children, which found that giving fluid boluses to severely ill children with impaired perfusion in resource-limited settings in Africa actually increased mortality, was a clear demonstration of the potential dangers of plausible extrapolation [7]. All patients, irrespective of the location and circumstances of their illness, deserve evidence-based care. Yet, when we examine recent notable outbreaks including severe acute respiratory syndrome (SARS), avian influenza, pandemic influenza, MERS-CoV and EVD, very few patients have benefited from clinical research. Indeed we have yet to identify an effective therapeutic agent for any of these infections. Whilst the neuraminidase inhibitors (e.g. oseltamivir) have demonstrated efficacy in shortening symptom duration in uncomplicated influenza and as prophylactic agents, uncertainty remains as to their effectiveness in preventing and treating severe influenza [8–10]. Despite almost 800 avian influenza A/H7N9 cases since 2013, there is only one registered treatment trial on clincialtrials.gov (NCT02095444). This represents a major global vulnerability, given that both the probability and impact of an influenza pandemic are high, and pandemic influenza vaccines cannot yet be produced in a timeframe to impact the first wave of the pandemic. There are also only two clinical treatment trials registered for MERS Co-V (NCT02845843, NCT02190799), although cases have now been reported for 4 years. SARS provides an excellent example of the consequences of an inadequate clinical research response. The antiviral ribavirin was widely used during the early outbreak owing to its broad action and prior experience with its use for other indications. As the epidemic progressed, small case series and emerging in vitro data suggested poor efficacy and tolerability, and so use of this agent declined [11]. However, retrospective review of these series indicated they had significant methodological limitations [12], and if another SARS outbreak was to occur there remains no clear consensus on ribavirin use. There has also been a failure to gather evidence on the effectiveness of readily available and widely used supportive care measures. For example, when treating EVD there remains no robust evidence on the optimal intravenous fluid resuscitation strategy, the use of vitamin K, or the provision of loperamide for diarrhoea, all practices that were adopted to varying extents during the West Africa (2013–2016) epidemic.

#### Helping to control the epidemic

**Fig. 1:**
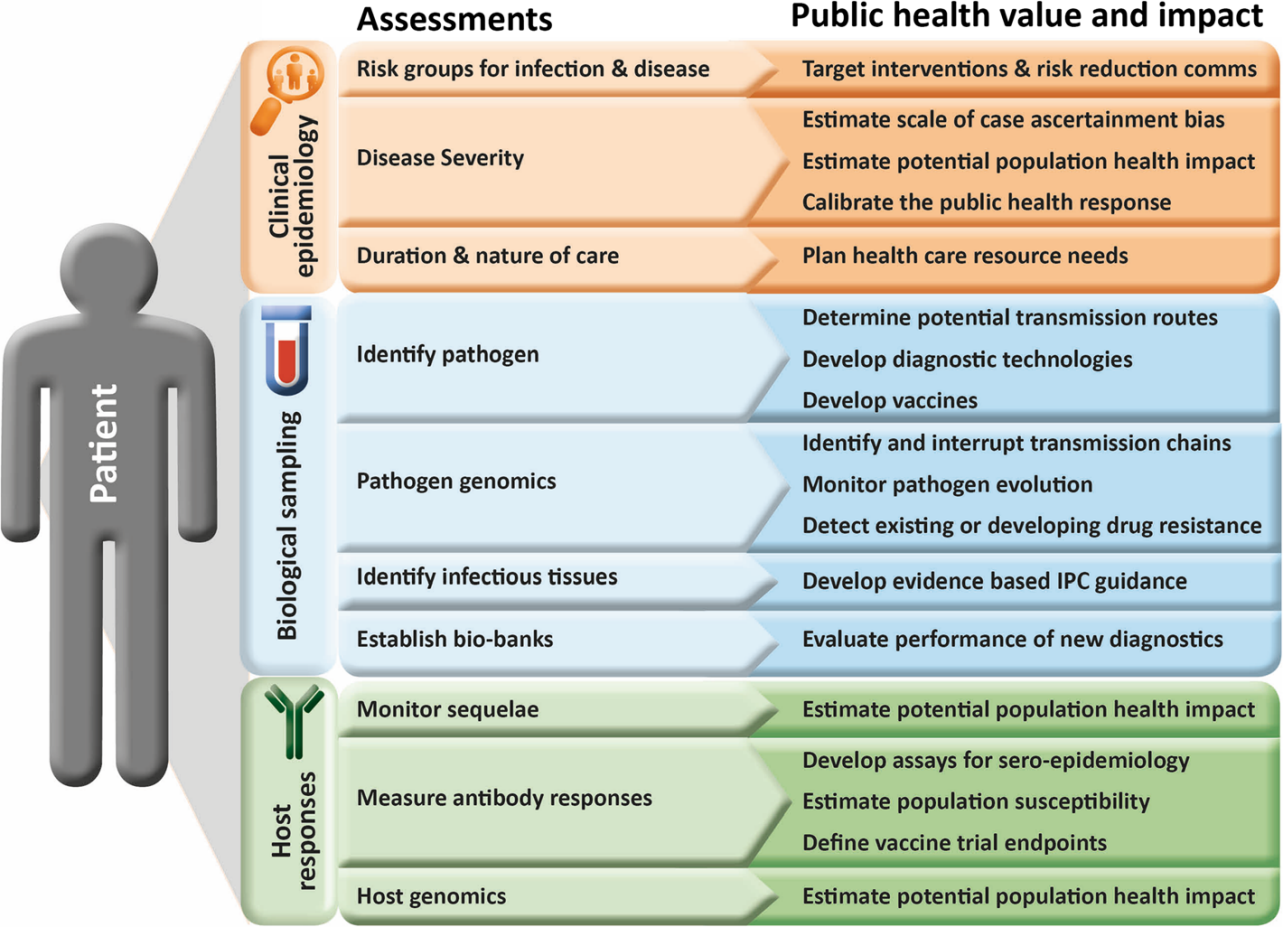
The public health value and impact of patient-centred assessments

Patients with epidemic and emerging infections deserve to benefit from the fruits of research as much as any other patient, yet the broader societal benefits of clinical research are even greater in the context of outbreaks. A well-focused and calibrated public health response to an epidemic can save lives and money. The West Africa Ebola epidemic is set to become a notorious case study of the consequences of under-reaction, whereas the early response to the 2009 A/H1N1 influenza pandemic is widely considered to have been an over-reaction [13]. Many aspects of an appropriate public health response are dependent on high-quality data and samples from patients. For example, reliable illness severity data are required to predict the number of infected and ill people and then scale the response appropriately; groups at high risk for infection or poor prognosis need to be identified for targeted preventative and treatment interventions; genetic sequencing of pathogens from biological samples can provide critical information on transmission pathways, evolutionary pressures and drug resistance; and the characterisation of immunological responses is a prerequisite for developing the laboratory tools for critical sero-epidemiologic and vaccine immunogenicity studies. Figure 1 summarises the public health value of some of the key parameters that can only be derived from patients.

Even when faced with an outbreak of what we think is a well-characterised infection, there is always a need to critically re-evaluate received wisdom and to be sceptical of initial impressions. Influenza is a good case in point. The initial public health response to the 2009 influenza A/H1N1 pandemic is widely considered to have been poorly calibrated owing to excessive early estimates of the case fatality rate based on data from Mexico City and Winnipeg [14, 15]. What was initially thought to be a severe novel influenza turned out to be no more severe than an average seasonal influenza [16]. The resulting expenditure on antivirals and vaccines has been widely criticised and illustrates the social and economic imperative for a rigorous approach to assessing disease severity that explicitly considers biases that are inherent in surveillance and reporting systems.

As a result of the limited availability of systematically collected natural history data for EVD, it has only recently been established that fever is absent in approximately 10% of patients [17, 18]. However, fever was used for the entirety of the West Africa epidemic as often the solitary indicator for screening at airports and checkpoints, and as part of the case definition for Ebola virus testing [19, 20]. Limitations in collecting and analysing biological specimens from patients have resulted in inadequate understanding of transmission risks. For example, despite 22 prior EVD outbreaks and around 2000 cases, it was also only in 2015 that the risk of sexual transmission was confirmed [21]. There have been few, if any, comparable and comprehensive sampling studies done for other high-threat epidemic-prone diseases, even those with predictable seasonal outbreaks such as Crimean-Congo haemorrhagic fever. During the most recent Public Health Emergency of International Concern, the Zika virus outbreak, the poor availability of well-characterised patient-derived samples has impeded the development and validation of crucial assays for patient diagnosis [22].

It is clear that there is significant room for improvement in the systematic collection of data and biological samples from patients with the explicit aim of improving the evidence-base for public health decision-making.

### How do we make progress?

#### Integrated clinical data capture

**Fig. 2:**
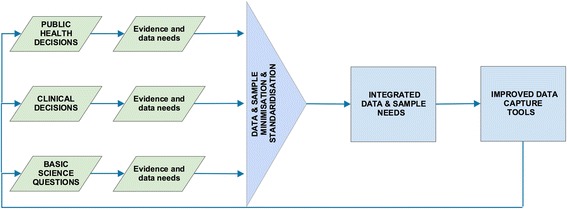
A schematic for interdisciplinary decision-driven data and sample collection

Currently, outbreak response is characterised by an artificial separation of the public health, clinical and scientific response. This is an understandable consequence of engrained disciplinary divisions and regulatory frameworks but is inefficient given that the ultimate aims of all groups are to improve patient outcomes and control the epidemic. Under even a cursory examination, it is clear that the boundaries between the public health, clinical and scientific response are blurred, with the necessary evidence overlapping and being collected from the same patient. What distinguishes research from clinical or public health practice is often difficult to define, and rather than trying to draw arbitrary boundaries, we should aim to integrate the data needs of all disciplines. The quality of evidence could then be improved by designing unified data and sample collection protocols that are driven by an explicit link to the public health and clinical decisions that need to be taken. Rather than using inferential techniques such as mathematical modelling to compensate for suboptimal data, analysis-ready datasets that provide meaningful information to support individual and population-based practices could be generated by working forwards from a suite of public health and clinical decisions, to evidence needs, and finally to data needs (Fig. 2). The goal of such a decision-driven data collection approach is to identify the most efficient way to improve the precision and timeliness of pivotal estimates such as attack rate, case-fatality rate, transmission probability and infectivity, whilst simultaneously providing the data elements that are needed for bedside clinical decision-making. This approach should include prior consideration of the impact of missing data and sampling biases on the validity, accuracy, and precision of estimates.

Whilst it would be naïve to think there are template data capture tools or protocols that will perfectly fit any outbreak, it would be a major step forward if data capture instruments were to be developed that are explicit about the content, quality and scale of data needed to take decisions, combined with a risk-based, rather than a discipline-based, approach to the need for ethical review and consent. Because outbreaks involve a complex interaction between the rights, responsibilities, benefits and risks of both individuals and communities, there will often be a need for careful community engagement and for ethical review by committees that are well versed in the specific ethical issues raised by epidemics [23].

#### New clinical study methodologies and tools

There is no doubting that epidemics are a challenging context in which to conduct research, but that just means we must adapt, not abandon, our research approach. Significant improvements in the care provided for patients treated for battlefield trauma [24] and in pre-hospital settings [25, 26] over the last few decades demonstrate that it is feasible to conduct patient-centred research in austere and challenging environments. The biggest remaining challenge for clinical research on EEIDs is uncertainty: emerging infections are often relatively rare; understanding of the clinical presentation and natural history is usually limited; and outbreaks are unpredictable in timing, location and size. Classical clinical trial designs that require predictable and often large case numbers to test hypotheses are not well suited to this epidemiological uncertainty. Trial designs are needed that are robust to uncertainties in the number, timing and location of cases; clinical phenotype, progression and outcomes; the optimal comparison (control) group; and the optimal intervention to test. The West Africa Ebola outbreak stimulated some innovative thinking in the design of clinical trials [27, 28] and this needs to continue. It should encompass designs for descriptive clinical studies, and prophylactic, therapeutic and vaccine trials under a variety of epidemiological scenarios.

The successful implementation of protocols for integrated clinical data collection and of novel clinical trials will require action to lower the barriers to the collection and sharing of standardised data. Such action should include the development of standardised data libraries and therapeutic area standards for epidemic infections (using the Clinical Data Standards Consortium system, cdisc.org); designing and evaluating generic disease severity scores and outcome measures; and the development of user-friendly, scalable and open-access software for data capture and sharing (including the use of federated databases).

#### Strengthened global coordination and support for clinical research on EEIDs

It is worth considering the substantial difficulties that face those who wish to undertake clinical research on emerging and epidemic infections. First, many emerging pathogens might be considered rare. The European definition of a ‘rare disease’ is a disease that affects fewer than 1 in 2000 people, whereas the US definition is a disease that affects fewer than 200,000 citizens [29]. The diseases currently under the ‘rare diseases’ umbrella are largely severe non-communicable diseases with a genetic component, such as cystic fibrosis, or rare cancers. Although direct comparison of infectious epidemic diseases and rare non-infectious diseases is somewhat artificial, it can nevertheless be illustrative. SARS resulted in a total of 8096 cases [30]; 850 cases of avian influenza A/H5N1 have been reported since 2003 [31]; 684 cases of avian influenza A/H7N9 have been reported since March 2013; and 1733 MERS-CoV cases have been reported since September 2012 [32]. For comparison there are an estimated 14,000 people living with phenylketonuria and around 225 new diagnoses of Ewing sarcoma annually in the US alone. Second, the timeframe for action can be both unpredictable and extremely short, with the average duration of influenza epidemics being 10 weeks, with the peak incidence reached after only 4 weeks [33]. Third, the spatial distribution can be widespread. The 660 patients diagnosed with avian influenza A/H7N9 in China between March 2013 and September 2015 were admitted to 258 different hospitals, an average of under one patient per hospital per year (personal communication, Yu Hongjie, China CDC). The 854 H5N1 cases reported since 2004 have arisen in 16 different countries [34].

The bottom line is that the unpredictability, rapidity and rarity of many emerging infectious disease outbreaks render it improbable that a meaningful research response can be delivered by isolated investigators or institutions. Large-scale international collaboration is essential. In the wake of the West Africa Ebola outbreak, several initiatives have highlighted and attempt to address key deficiencies in our ability to respond to major infectious disease outbreaks. These include the newly established WHO Health Emergencies Programme, the WHO R&D Blueprint for Action to Prevent Epidemics, the report of the Commission on a Global Health Risk Framework for the Future, and the Coalition for Epidemic Preparedness Innovations. However, none of these initiatives specifically or adequately address the weaknesses of platforms for conducting essential clinical research both before and during outbreaks. This renewed interest in global health security and in research and development for epidemic infections is to be welcomed but must be accompanied by investment in a sustainable operating model for EEID clinical research networks. Otherwise the clinical research platforms and tools that are needed to rapidly characterise emerging infectious threats and to evaluate the products of diagnostic, drug and vaccine development pipelines will, once again, not be there when we need them.

One of the earliest clinical research networks with a specific focus on EEIDs was the South-East Asia Infectious Diseases Clinical Research Network (SEAICRN), which was established in response to the re-emergence of avian influenza A/H5N1 in 2003. Following from SEAICRN, the International Severe Acute Respiratory and Emerging Infections Consortium (ISARIC) was established in 2012 as a global investigator-led network-of-networks aiming to ‘foster global collaborative patient-oriented research between and during epidemics’ [35]. ISARIC members have subsequently been prominent in the development of two further regional clinical research networks focused on preparedness for emerging and epidemic infections: the European Commission-funded Platform for European Preparedness Against (Re-) emerging Epidemics (PREPARE) and the Australian Partnership for Preparedness Research on Infectious Disease Emergencies (APPRISE). These networks have made significant contributions to building capacity [36], linking researchers, developing tools such as syndrome-based clinical characterisation and generic treatment trial protocols [35, 37, 38], identifying ethical and legal barriers [39], and responding to outbreaks [40–48].

However, sustaining and coordinating EEID clinical research networks is a major challenge when both disease incidence and funding are unpredictable and fluctuating. It simply is not realistic to establish and maintain epidemic clinical research capabilities in every centre where an outbreak might occur. This is particularly true in areas where poverty and inadequate healthcare systems mean that despite increased vulnerability to epidemic infections there are far more pressing day-to-day priorities. This does not mean that the only answer is to parachute researchers into an affected area. A model that has worked well for rare non-communicable diseases is the establishment of Rare Diseases Clinical Research Consortia, which are supported by a Rare Diseases Data Management and Coordination Center. This may be a good model for EEIDs, where geographic or disease-specific clinical research networks working on day-to-day infectious diseases problems (including drug-resistant infections) are primed and incentivised to respond to outbreak infections, and are supported by a centre of excellence that houses the expertise and resources required to develop and test new methods and tools, to coordinate or lead multi-centre research on EEIDs, and to provide much needed support and tools to local investigators in the event of an emergency. This might be conceptualised as a multiple hub-and-spoke, or dandelion, model, where each research network has its own hub, but each hub can call on the support and resources of a central centre of excellence.

### Summary

The response to epidemics has been plagued with poor data and weak evidence, and the central importance of patient-based clinical research is widely underappreciated. We risk continuing to fail the patients and communities most affected unless we work towards an improved framework. Key features of this improved framework include integrating patient-centred research with other aspects of outbreak response, developing methods and tools that address the very real epidemiological and contextual challenges of EEIDs, and building an organisational model for clinical research on EEIDs that is effective and sustainable.

## Abbreviations

EEID: Emerging and epidemic infectious diseases
EVD: Ebola virus disease
ISARIC: International Severe Acute Respiratory and Emerging Infection Consortium
MERS CoV: Middle East respiratory syndrome coronavirus
SARS: Severe acute respiratory syndrome
WHO: World Health Organization
